# Shortwave Infrared-Emitting Theranostics for Breast Cancer Therapy Response Monitoring

**DOI:** 10.3389/fmolb.2020.569415

**Published:** 2020-10-06

**Authors:** Jay V. Shah, Amber Gonda, Rahul Pemmaraju, Aishwarya Subash, Carolina Bobadilla Mendez, Marissa Berger, Xinyu Zhao, Shuqing He, Richard E. Riman, Mei Chee Tan, Mark C. Pierce, Prabhas V. Moghe, Vidya Ganapathy

**Affiliations:** ^1^Department of Biomedical Engineering, Rutgers University, Piscataway, NJ, United States; ^2^Engineering Product Development, Singapore University of Technology and Design, Tampines, Singapore; ^3^Department of Materials Science and Engineering, Rutgers University, Piscataway, NJ, United States; ^4^Department of Chemical & Biochemical Engineering, Rutgers University, Piscataway, NJ, United States

**Keywords:** tumor therapy monitoring, rare earths, shortwave infrared imaging, nanotechnology, theranostic, NIR-II bioimaging

## Abstract

Therapeutic drug monitoring (TDM) in cancer, while imperative, has been challenging due to inter-patient variability in drug pharmacokinetics. Additionally, most pharmacokinetic monitoring is done by assessments of the drugs in plasma, which is not an accurate gauge for drug concentrations in target tumor tissue. There exists a critical need for therapy monitoring tools that can provide real-time feedback on drug efficacy at target site to enable alteration in treatment regimens early during cancer therapy. Here, we report on theranostic optical imaging probes based on shortwave infrared (SWIR)-emitting rare earth-doped nanoparticles encapsulated with human serum albumin (abbreviated as ReANCs) that have demonstrated superior surveillance capability for detecting micro-lesions at depths of 1 cm in a mouse model of breast cancer metastasis. Most notably, ReANCs previously deployed for detection of multi-organ metastases resolved bone lesions earlier than contrast-enhanced magnetic resonance imaging (MRI). We engineered tumor-targeted ReANCs carrying a therapeutic payload as a potential theranostic for evaluating drug efficacy at the tumor site. *In vitro* results demonstrated efficacy of ReANCs carrying doxorubicin (Dox), providing sustained release of Dox while maintaining cytotoxic effects comparable to free Dox. Significantly, in a murine model of breast cancer lung metastasis, we demonstrated the ability for therapy monitoring based on measurements of SWIR fluorescence from tumor-targeted ReANCs. These findings correlated with a reduction in lung metastatic burden as quantified via MRI-based volumetric analysis over the course of four weeks. Future studies will address the potential of this novel class of theranostics as a preclinical pharmacological screening tool.

## Introduction

Treatment of cancers through conventional chemotherapy or targeted therapy is challenging due to the heterogeneity of drug concentration in tumors as a result of varied tissue penetration and imperfect vasculature, which may lead to stasis of blood flow, large inter-capillary distances, and raised interstitial pressure (Tannock, 1998). Hence, the use of mean drug concentration is often misleading as a therapeutic index for drug efficacy. Current therapeutic drug monitoring (TDM) tools offer a systemic outcome, such as a measure of a surrogate drug concentration in the plasma, which correlates with the overall outcome of tumor regression, but they fail to provide local drug concentrations at the lesion site. Additionally, resistance to chemotherapy regimens requires timely decisions for treatment alteration for improved disease outcomes (Kulkarni et al., 2016; Onafuye et al., 2019). Cancer treatment currently faces two major challenges. First, there is a need for effective drug delivery tools that can enhance bioavailability at the tumor site while reducing non-specific interactions with off-target healthy organs (Kantamneni et al., 2017; Senapati et al., 2018; Bartelink et al., 2019). Second, the assessment of therapeutic outcomes requires a reliable TDM tool that can inform on drug pharmacokinetics at target site.

Nanoparticle delivery systems, such as liposomal, protein-based (e.g., albumin), and polymeric, have been shown to reduce systemic toxicity compared to free drug, as well as improved target site delivery, leading to increased drug concentration at the tumor site (Senapati et al., 2018; Anselmo and Mitragotri, 2019). Albumin, in particular, as either a drug conjugate (Cranmer, 2019) or nanocarrier (An and Zhang, 2017), can improve circulation time of the drug (Karami et al., 2020), facilitate increased tumor tissue accumulation via the enhanced permeability and retention (EPR) effect (Kimura et al., 2018), and overcome drug resistance (Onafuye et al., 2019). Adapting an effective delivery tool to address the lack of TDM has fueled recent research into a new field of theranostics.

Theranostics, the potential to combine an effective therapeutic with a diagnostic agent for therapy monitoring, has thus far been explored primarily using nuclear agents both in preclinical and clinical studies (Nock et al., 2017; Yu et al., 2018; Erdmann et al., 2019). In 2018, the United States Food and Drug Administration approved the theranostic Lutathera^®^, a Lutetium (Lu) 177-conjugated somatostatin receptor-targeting drug for neuroendocrine tumors (Hennrich and Kopka, 2019). However, the use of radionuclides as a theranostic tool is challenging since the exposure of the excreted radionuclide and the half-life of decay for the agent need to be balanced with the therapeutic effect of the drug (Turner, 2018, 2019). Furthermore, nuclear imaging is limited by high costs, lengthy scan times, and the use of radiotracers and ionizing radiation.

Optical imaging is a relatively simple and safe alternative platform that can be used for therapy monitoring without the need for ionizing radiation (Zavaleta et al., 2018; Fiordelisi et al., 2019). Optical imaging provides excellent sensitivity, with the ability to image at molecular resolution (Wang et al., 2015). However, *in vivo* optical imaging is limited due to a shallow millimeter-scale depth of penetration and tissue autofluorescence in the visible spectrum (λ = 400–700 nm) (Cao et al., 2019). Previously, we have addressed this shortcoming of optical imaging through the use of rare earth (Re)-doped ceramic nanoprobes that emit shortwave infrared (SWIR) light (λ = 1000–1700 nm) following excitation by a 980 nm source (Naczynski et al., 2010). SWIR, also referred to as the second optical window of near infrared light (NIR-II), can penetrate deeper into tissue because of lower absorbance than visible light (Cao et al., 2019). Furthermore, reduced scattering and autofluorescence compared to both visible and NIR-I wavelengths (λ = 700–1000 nm) results in significantly improved signal-to-noise ratios without a loss of spatial resolution (Cao et al., 2019). Naczynski et al. designed a biocompatible formulation of Re-doped ceramic nanoprobes through encapsulation in human serum albumin to generate rare earth albumin nanocomposites (ReANCs) and subsequently established these as novel diagnostic agents in tumor surveillance (Naczynski et al., 2013). The unique property of albumin, namely, the presence of drug binding pockets (Zsila et al., 2011; Abou-Zied, 2015) and functional groups (Frei, 2011; Zsila et al., 2011; Abou-Zied, 2015; Liu and Chen, 2016), renders ReANCs as promising theranostic candidates. ReANCs have been loaded with therapeutic payloads (Cui et al., 2013) or functionalized with targeting ligands (Zevon et al., 2015; Kantamneni et al., 2017). Functionalization of ReANCs with the small molecule AMD3100, an antagonist of tumor-expressed C-X-C chemokine receptor type 4 (CXCR4), showed higher retention in CXCR4-positive tumors, allowing for detection of sub-tissue microlesions as small as 18.9 mm^3^ and about 1 cm deep (Zevon et al., 2015). Furthermore, in a longitudinal study, SWIR emissions from ReANCs were used to monitor the progression of multi-organ metastases and most significantly, detect micro-lesions in the bone earlier than contrast-enhanced MRI (Kantamneni et al., 2017). Theranostic agents capable of optical imaging have been explored for therapy monitoring in subcutaneous models of breast cancer with moderate success (Ma et al., 2019). However, an optical theranostic agent that can monitor tumor metastases has not been studied to date.

**FIGURE 1:**
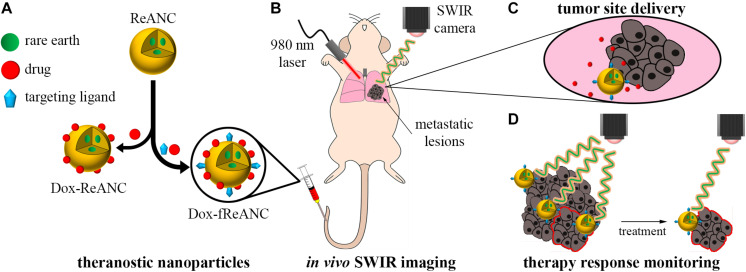
Therapeutic drug monitoring by SWIR-emitting rare-earth albumin nanocomposites. Human serum albumin encapsulated SWIR-emitting rare earth-doped nanoparticles (ReANCs) were synthesized by controlled coacervation. The targeting ligand (AMD3100) and/or the drug (doxorubicin) were physically adsorbed sequentially onto the surface of ReANCs **(A)**. SWIR imaging was used to detect lung metastatic lesions in a mouse model of triple negative breast cancer metastasis **(B)**. Tumor-targeting fReANCs facilitated delivery of doxorubicin to the lesion site **(C)**. SWIR emissions from the theranostic particles retained in the lungs were used as a metric of therapy response that correlated with tumor progression **(D)**.

**FIGURE 2:**
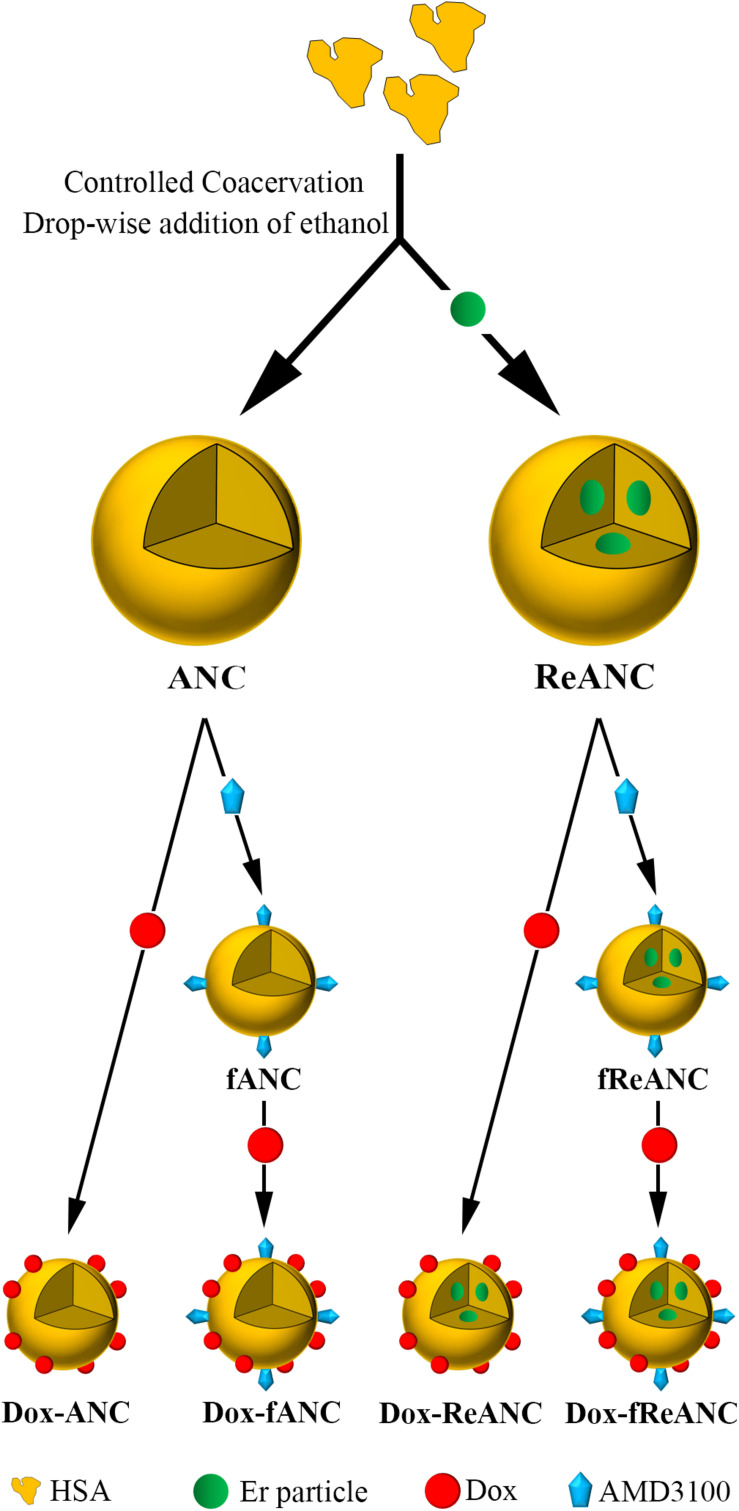
Design and fabrication of albumin-based nanocarriers and theranostic nanoparticles. Albumin nanocomposites (ANCs) were synthesized via controlled coacervation by adding pure ethanol drop-wise to a solution of human serum albumin. Dissolving Er-doped nanoparticles in the ethanol prior to synthesis resulted in encapsulation of the nanoprobes in albumin, producing diagnostic ReANCs. Tumor-targeting particles (fANCs and fReANCs) were synthesized following physical adsorption of the CXCR4 antagonist AMD3100, which improved uptake of the particles by CXCR4-expressing cancer cells. Doxorubicin (Dox) was then loaded to both tumor-targeting and non-targeting albumin particles by physical adsorption, producing therapeutic particles. ReANCs loaded with Dox (Dox-ReANCs and Dox-fReANCs), possessing both diagnostic and therapeutic components, served as theranostic agents.

In this study, we investigated both the ability of ReANCs to deliver a therapeutic payload to the tumor site and the efficacy of ReANCs to track tumor regression in response to treatment. We report for the first time a SWIR-based optical imaging approach to breast cancer lung metastasis therapy monitoring via theranostic particles in a mouse model of human breast cancer (Figure 1). The model chosen was triple negative breast cancer (TNBC), which lacks the expression of targetable hormone receptors or HER2 (Waks and Winer, 2019) and thus remains largely refractory to chemotherapy. We generated tumor-targeted theranostic particles by sequential physical adsorption of a targeting ligand, AMD3100, and drug, doxorubicin (Dox), to the surface of ReANCs (Figure 2) and showed, through *in vitro* cellular uptake studies, specificity and effective anti-tumor pharmacological activity of the theranostic particles on the TNBC cell line, MDA-MB-231. Albumin nanocomposites (ANCs) without rare earth cores encapsulated within them (Naczynski et al., 2010) were used to validate the drug release profiles and cytotoxic capability of theranostic particles *in vitro*. We found the Dox release profiles to be consistent with previously published studies (Lin et al., 2017; Bi et al., 2018) and pH-dependent. Further, we demonstrated that functionalized particles (fANCs or fReANCs) improved the delivery of Dox to cancer cells *in vitro* through cellular uptake studies. Finally, in a proof-of-concept mouse model of lung metastasis of human TNBC, we demonstrated that the inclusion of Dox as payload on tumor-targeted fReANCs provided real-time therapy monitoring along with a better therapeutic outcome in mice after four weeks of treatment than Dox adsorbed onto non-targeted ReANCs. Our findings from SWIR imaging were validated and correlated with tumor volumes measured by conventional MRI. We conclude that the use of SWIR-emitting theranostic particles for TDM in a preclinical setting can provide a pharmacological screening platform to assess the therapeutic outcomes of different treatment regimens.

## Materials and Methods

### Synthesis of Rare Earth Albumin Nanocomposites (ReANCs)

Rare earth (Re) nanoparticles consisting of NaYF_4_ cores doped with ytterbium (Yb) and erbium (Er) encased by NaYF_4_ shells were synthesized by burst nucleation reaction as described previously (Naczynski et al., 2013; Zhao et al., 2016). Re nanoparticles were encapsulated in human serum albumin (HSA) via solvent-induced controlled coacervation as previously reported (Naczynski et al., 2013; Zevon et al., 2015; Kantamneni et al., 2017). Briefly, Re nanoparticles were dissolved in pure ethanol at a concentration of 0.8 mg/mL and sonicated for 1 h. HSA (Sigma-Aldrich, St Louis, MO) was dissolved in 10 mM NaCl at a concentration of 20 mg/mL and adjusted to a pH of 8.50 ± 0.05 with 0.1 M NaOH. Using a syringe pump set to an infusion rate of 1.5 mL/min, 2 mL of Re in ethanol was added to 500 μL of the pH-adjusted HSA solution under continuous stirring at 850 RPM at room temperature. Following the addition of ethanol to the HSA, 2.34 μL of glutaraldehyde (Sigma-Aldrich, St. Louis, MO) was added to the suspension to allow cross-linking for 16–18 h in order to form ReANCs. ReANCs were purified with three cycles of centrifugation (Avanti J-E centrifuge, Beckman Coulter, Brea, CA) for 10 min at 4°C at 48,400 × *g* and finally suspended in PBS to obtain a 10x concentration (encapsulated Re concentration of 8 mg/mL and total albumin concentration of 50 mg/mL). The same procedure was followed in the synthesis of albumin nanocomposites (ANCs) devoid of Res, with the exception of using pure ethanol during coacervation instead of Re particles dissolved in ethanol and a stir rate of 1000 RPM.

### Loading Doxorubicin Onto ANCs and ReANCs [Dox-(Re)ANCs]

Doxorubicin HCl (MedKoo Biosciences, Morrisville, NC) was dissolved in DMSO. The drug was physically adsorbed to the surface of ANCs and ReANCs by adding 388 μL of Dox solution (0.4–1.0 mg/mL, dependent on target concentration) to 3.5 mL of (Re)ANCs (1x concentration, i.e., total albumin concentration of 5 mg/mL), followed by constant agitation at room temperature for 3 hours. Excess Dox was removed by centrifugation. Subsequently, the amount of Dox loaded onto particles was evaluated by measuring Dox fluorescence (Ex: 470 nm, Em: 595 nm) in the supernatant using a microplate reader (Tecan, Switzerland). The concentration of Dox in the supernatant was calculated against a Dox standard curve. The loading efficiency (LE) of Dox was assessed based on the fluorescence of unloaded Dox in the supernatant (Janes et al., 2001; Andhariya et al., 2010; Qi et al., 2010; Nigam et al., 2011; Abbasi et al., 2012; Park et al., 2012; Unsoy et al., 2014; Angelopoulou et al., 2019) using the equation:

$$L\,E=\frac{a\,m\,o\,u\,n\,t\,o\,f\,D\,o\,x\,l\,o\,a\,d\,e\,d-a\,m\,o\,u\,n\,t\,o\,f\,D\,o\,x\,u\,n\,l\,o\,a\,d\,e\,d}{a\,m\,o\,u\,n\,t\,o\,f\,D\,o\,x\,l\,o\,a\,d\,e\,d}\times 100\%$$

### Functionalization of ANCs and ReANCs With AMD3100

ANCs and ReANCs were functionalized with AMD3100 as previously described (Zevon et al., 2015; Kantamneni et al., 2017). Briefly, AMD3100 (EMD Millipore, Burlington, MA) was dissolved in sterile water at a concentration of 1.25 μM and adsorbed onto nanoparticles by adding 388 μL of the AMD3100 solution to 3.5 mL of ANCs or ReANCs (1x concentration, i.e., total albumin concentration of 5 mg/mL), followed by constant agitation at room temperature for 3–4 h. This concentration of AMD3100 was found to be the optimal loading condition based on previously described *in vitro* experiments (Zevon et al., 2015). For particles that were dual loaded with AMD3100 and Dox (Dox-fANCs or Dox-fReANCs), AMD3100 functionalization was performed prior to Dox loading.

### Characterization of ANCs and ReANCs

The nanoparticle yield was determined by performing a bicinchoninic acid (BCA) assay (Thermo Fisher Scientific, Waltham, MA) according to manufacturer’s instructions with the supernatant obtained during the purification process. Particle yield was found to be ∼80%. Nanoparticle size and polydispersity were assessed via dynamic light scattering (DLS) with a ZetaSizer (Malvern Panalytical, United Kingdom).

### Cell Culture

MDA-MB-231 cells were obtained from American Type Culture Collection (ATCC, Old Town Manassas, VA) and cultured in Dulbecco’s Modified Eagle Medium (DMEM) (Gibco, Waltham, MA) supplemented with 10% fetal bovine serum (FBS) (Thermo Fisher Scientific, Waltham, MA) and 1% penicillin-streptomycin (Thermo Fisher Scientific, Waltham, MA) at 37°C in a humidified atmosphere of 5% CO_2_.

### Cell Viability Assay

MDA-MB-231 cells were plated in triplicate at a density of 1 × 10^4^ cells per well in a 96 well plate and allowed to attach overnight (*n* = 3 per sample). To determine the toxicity of Dox-ANCs over time, cells were washed with PBS, and fresh media containing Dox-ANCs or free Dox at an equivalent concentration of 0.02 mg/mL was added to the cells. The negative controls for each time point were cells treated with fresh media without Dox or Dox-ANCs. At each time point (0, 1, 2, 4, 8, 12, 24, 48, and 72 h), media was removed, cells were washed twice with PBS to remove residual drug from the wells, and viability was assessed using a 3-(4,5-dimethylthiazol-2-yl)-2,5-diphenyltetrazolium bromide (MTT) assay (Thermo Fisher Scientific, Waltham, MA) according to manufacturer’s instructions. Briefly, wells were replaced with 100 μL of fresh culture media. Each well received 10 μL of 12 mM MTT solution, and the cells were incubated at 37°C for 4 hours. Following incubation, 100 μL of sodium dodecyl sulfate (SDS) prepared in 0.01 M HCl at a concentration of 0.1 g/mL was added to each well, mixed thoroughly, and incubated at 37°C for 4 h. The absorbance from the wells was read at 570 nm using a microplate reader (Tecan, Switzerland).

### Dox Release From ANCs

Release of Dox from nanoparticles was assessed by microdialysis (Michalowski et al., 2004). Dox-ANCs, Dox-fANCs, Dox-ReANCs, or Dox-fReANCs (50 μl) were loaded into a microplate dialysis device with a 10,000 molecular weight cutoff (Thermo Fisher Scientific, Waltham, MA) and placed into a 5 mL centrifuge tube containing 1 mL of release buffer. Release buffer consisted of PBS adjusted to pH 7.4, pH 6.5, or pH 5.0 in order to simulate different physiological conditions. The centrifuge tubes were closed and incubated at 37°C in a humidified atmosphere of 5% CO_2_. At each designated time point (0, 1, 2, 4, 8, 12, 24, and 48 h), the dialysis device was removed and placed into a new centrifuge tube containing fresh release buffer. The Dox fluorescence from the buffer (Ex: 470 nm, Em: 595 nm) was measured to determine the amount of Dox released from the nanoparticles. The cumulative release profile was plotted against time. Release studies were performed three times, and the fluorescence from each sample was read in triplicate.

### *In vitro* Dox Uptake

Cellular uptake of Dox by MDA-MB-231 cells treated with Dox-ANCs and Dox-fANCs was assessed with a Zeiss LSM 780 confocal microscope (Zeiss, Germany). Cells were seeded at a density of 2.5 × 10^4^ cells per well in a 96 well plate and allowed to attach overnight. Cells were treated with ANCs, fANCs, Dox-ANCs, Dox-fANCs, or free Dox at an equivalent Dox concentration of 0.02 mg/mL for 8 h. Cells were fixed with 4% paraformaldehyde and stained to visualize DNA using Hoechst 33342 (Thermo Fisher Scientific, Waltham, MA). The natural fluorescence of Dox was imaged in relation to the cell nucleus using a 488 nm laser line for Dox and a 405 nm laser line for Hoechst 33342. Images were captured from three distinct locations per well from a total of three wells per group. Images were analyzed with open source Fiji software^1^ to determine the signal intensity normalized to a manual cell count.

### Pharmacodynamics Analysis

Western blots were performed to validate the Dox mechanism of action in Dox-ANCs *in vitro* and Dox-ReANCs *in vivo*. For *in vitro* studies, MDA-MB-231 cells were treated with Dox-loaded ANCs and subsequently lysed. For *ex vivo* studies, fresh frozen lung tissue from non-tumor bearing mice and lungs from untreated and Dox-treated tumor bearing mice were homogenized using a mortar and pestle. Homogenates and cells were lysed with RIPA buffer and phosphatase inhibitors. Total protein concentration was determined using a BCA assay. A total of 30 μg of protein was electrophoresed using pre-cast 4–12% Bis-tris (Thermo Fisher, Waltham, MA) SDS PAGE gels and transferred to PVDF membranes. The membranes were blocked with 5% milk for 1 h at room temperature. They were then incubated overnight at 4°C with primary antibody and an HRP-conjugated secondary antibody for 1 h at room temperature. SuperSignal Chemiluminescent HRP substrate (Thermo Fisher, Waltham, MA) was used according to the manufacturer’s instructions to detect the signals and image the bands. Antibodies used included rabbit anti-human Bcl-2 monoclonal antibody (Human Specific) (Clone D55G8; Cell Signaling, Danvers, MA) at 1:1000 dilution, rabbit anti-human cleaved PARP (Asp214) polyclonal antibody (Cell Signaling, Danvers, MA) at 1:1000 dilution, rabbit anti-human β-actin polyclonal antibody (Abcam, Cambridge, MA) at 1:3000, and goat anti-rabbit IgG HRP polyclonal antibody (Abcam, Cambridge, MA) at 1:5000 dilution.

### *In vivo* Model

All animal studies performed for this research were approved by the Institutional Review Board for the Animal Care and Facilities Committee of Rutgers University and performed in accordance with institutional guidelines on animal handling. Three- to four-week-old athymic nude mice were purchased from Charles River Laboratories (Fairfield, NJ) and housed in sterile disposable cages. Mice were provided with food and water *ad libitum* and allowed to acclimate for one week before beginning experimental procedures. For *in vivo* studies involving tumor-bearing animals, four- to five-week-old mice were injected with 3 × 10^5^ MDA-MB-231 cells intravenously by the tail vein. For proof-of-concept therapy monitoring studies, mice were divided into 5 groups: Dox-ReANCs, Dox-fReANCs, ReANCs, fReANCs, and healthy (non-tumor bearing) controls. After confirming formation of metastatic lesions in the lungs by MRI, mice in the treatment groups received weekly intravenous injections of 150 μL Dox-ReANCs or Dox-fReANCs at an equivalent dose of 2–2.5 mg/kg (drug/body weight) of Dox. Control mice received weekly intravenous injections of 150 μL ReANCs or fReANCs.

### SWIR Imaging and Analysis

An in-house small animal SWIR imaging system was used to image the animals as previously described (Naczynski et al., 2013; Zevon et al., 2015; Kantamneni et al., 2017; Higgins et al., 2018). Mice received anesthesia by inhalation of 2% isoflurane (Henry Schein Inc., Melville, NY) in oxygen at a flow rate of 1 L/min. Following injection of SWIR-emitting particles, the animals were continuously scanned with a collimated continuous-wave laser (980 nm wavelength, 9.6 mm beam diameter) at an intensity of 2.45 W/cm^2^ to excite the nanoparticles. Emitted SWIR light was detected with a 512 × 640 pixel InGaAs camera (640HSX, Sensors Unlimited, Princeton, NJ) equipped with two 1064 nm long-pass filters (Semrock, Rochester, NY), a 1497 – 1579 nm band-pass filter (Semrock), and a 25 mm fixed focal length, f/1.4 SWIR lens (StingRay Optics, Keene, NH). The system allows for real-time imaging with an exposure time of 33 ms per frame. Images were acquired as video files (.bin) during scanned illumination of the animal. Imaging was performed prior to administration of nanoparticles and again at 24 hours post-injection. White light images for each animal were taken prior to all SWIR imaging. A custom MATLAB script was implemented to convert the video files into a single image file (.tiff), containing the maximum value measured at each pixel over all frames acquired in the video. Quantitative analysis of the SWIR images was performed using a custom MATLAB graphical user interface (GUI). The pre-injection white light image, pre-injection SWIR image, post-injection white light image, and its corresponding post-injection SWIR image were opened with the GUI. Regions of interest (ROI) were manually selected based on anatomical landmarks visible in the white light images of the mice. These ROIs were then applied to the associated SWIR image, with the mean SWIR intensity within the pre-injection ROI subtracted from each individual pixel within the post-injection ROI. These background-corrected post-injection SWIR images were displayed as false-color overlays on the corresponding white light images.

### Magnetic Resonance Imaging

MRI was performed using a 1 tesla M2-High Performance MRI System (Aspect Magnet Technologies Ltd., Netanya, Israel). MRI procedures were performed with the mice under inhalation of 4% isoflurane in oxygen for induction of anesthesia and 1–2% isoflurane in oxygen for maintenance. The heart rate of the mice was monitored, and isoflurane exposure was adjusted to maintain a heart rate of 40–50 beats per minute. MRI images were analyzed using VivoQuant software (Aspect Magnet Technologies Ltd., Netanya, Israel) by performing volumetric analyses of manually selected regions of interest over detectable metastatic lesions.

### *Ex vivo* Analysis

At end point, mice were dosed with Dox-ReANCs, Dox-fReANCs, ReANCs, or fReANCs as determined by their group. Four hours post-injection, mice were euthanized by CO_2_ inhalation, followed by cervical dislocation according to institutional guidelines. A gross dissection was performed and the liver, spleen, kidneys, lungs, and heart were collected from each mouse. The excised organs were SWIR imaged with a collimated continuous-wave laser (980 nm wavelength, 9.6 mm beam diameter) at an intensity of 0.56 W/cm^2^ to excite the rare earth particles. Analysis of the SWIR emissions were performed using the post-processing GUI in MATLAB as described above, except a blank image was used as the pre-inject SWIR image. Organs were imaged (30 sec exposure; Ex: 480 nm, Em: 600 nm) using an *In Vivo* MS FX PRO optical imaging system (Bruker, Billerica, MA) to track the distribution of Dox. Fluorescent images were qualitatively analyzed using Fiji software.

### Statistical Analysis

All data is presented as the mean ± standard error of the means (SEM). For planning all *in vivo* studies, animal number was determined using power analysis (G^∗^Power 3.1.9.2, Heinrich Heine Universität Düsseldorf) with a statistical test (analysis of variance, one-way, omnibus) set to achieve 80% power with an effect size of 0.8 and α set to 0.05. Mice were randomly assigned to each experimental or control group, and investigators were not blinded to the groups. All statistical analyses were performed using Prism 8 software (GraphPad, San Diego, CA) and Origin 2020 software (Origin Lab, Northampton, MA). Statistical outliers were detected and removed from subsequent analysis using a Grubb’s test for outliers. Prior to statistical analysis, samples were screened for normality using a Shapiro-Wilk test for normality. A student’s *t* test was used to compare differences between two samples. When multiple samples were examined, an *F*-test of equality of variances with a confidence interval of 95% was performed, followed by a *post hoc* Tukey’s test to correct for multiple comparisons. When treatment and particle type were both compared as independent variables, a two-way analysis of variance (ANOVA) was performed, followed by a *post hoc* Tukey’s test to correct for multiple comparisons. A *p*-value less than 0.05 was considered statistically significant.

## Results

### Synthesis and Characterization of Nanoparticles

Theranostic human serum albumin encapsulated rare earth nanoparticles (ReANCs), capable of simultaneous lesion site drug delivery and therapy monitoring, were engineered as shown in Figure 2 (Naczynski et al., 2010, 2013; Cui et al., 2013; Zevon et al., 2015; Kantamneni et al., 2017). Prior studies established that cells respond similarly to both ReANCs and albumin nanocomposites without rare earth cores encapsulated within them (ANCs) (Naczynski et al., 2010). Therefore, ANCs with the therapeutic payload (Dox-ANCs) were used for pharmacokinetic studies *in vitro* (Supplementary Figure S1). Additionally, therapeutic ANCs (Dox-ANCs) were also of similar size to ANCs (data not shown).

**FIGURE 3:**
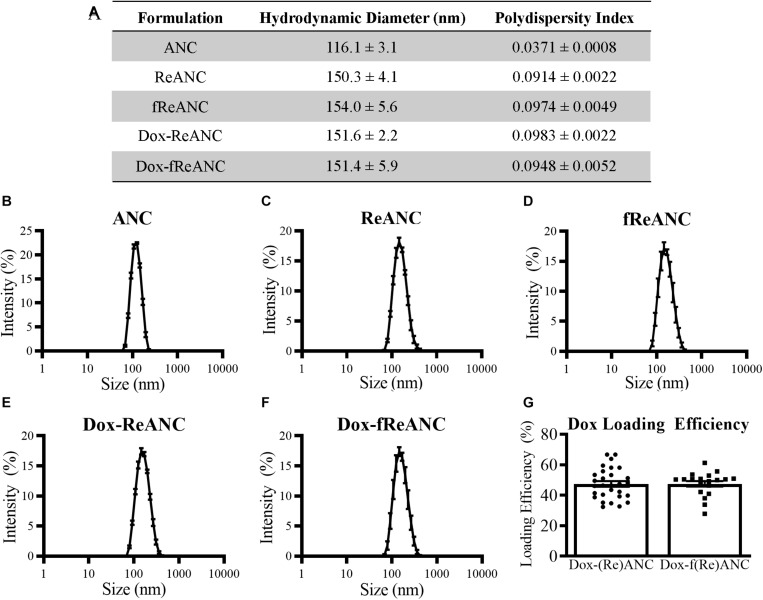
Characterization of ANC and ReANC formulations. The table **(A)** displays the hydrodynamic diameters and polydispersity indices (PDI) of ANCs **(B)**, ReANCs **(C)**, fReANCs **(D)**, Dox-ReANCs **(E)**, and Dox-fReANCs **(F)** were measured using dynamic light scattering. Data is presented as the mean ± SEM. All particle formulations had low polydispersity indices in solution. Functionalization with AMD3100 and Dox loading did not affect the size of ReANCs (*p* = 0.9535 assessed by an ordinary one-way ANOVA) or PDI (*p* = 0.3877). The loading efficiency of Dox adsorption onto the albumin shells of (Re)ANCs and f(Re)ANCs was calculated based on the fluorescence (Ex: 470 nm, Em: 595 nm) of unloaded Dox **(G)**. Loading efficiency was approximately 47% for both tumor-targeted and untargeted formulations, indicating that adsorption of AMD3100 for precision targeting prior to Dox loading did not affect the loading efficiency (*p* = 0.9833 assessed by a two-tailed student’s *t* test).

The ANCs used in this study were 116.1 ± 3.1 nm in diameter as assessed by dynamic light scattering (Figures 3A,B), while ReANCs measured 150.3 ± 4.1 nm, accounting for the encapsulation of ∼30 nm rare earth nanoparticle cores (Kantamneni et al., 2017), with a low PDI of 0.0914 ± 0.0022 (Figures 3A,C). ReANCs were functionalized (fReANCs) with the small molecule AMD3100 via physical adsorption as previously described (Zevon et al., 2015). fReANCs measured 154.0 ± 5.6 nm in diameter (Figures 3A,D) and were not significantly different than ReANCs in size.

For *in vivo* proof-of-concept therapy monitoring studies, we engineered theranostic particles with rare earth cores and therapeutic payloads, with or without targeting ligands. Previously, due to the presence of drug binding sites on albumin (Chen and Liu, 2016), ANCs have been used as drug delivery systems following adsorption of small molecule drugs such as riluzole (Cui et al., 2013). In this study, theranostic particles were engineered by physical adsorption of Dox to ReANCs (Dox-ReANCs) or tumor-targeted fReANCs (Dox-fReANCs). Dox-ReANCs and Dox-fReANCs were 151.6 ± 2.2 nm and 151.4 ± 5.9 nm, respectively (Figures 3A,E,F), indicating that loading of a therapeutic payload did not affect the physical characteristics of ReANCs (*p* = 0.95 for size and *p* = 0.39 for PDI as determined by a one-way ANOVA). LE was consistent between ReANCs and fReANCs (Figure 3G) with values of 47.49 ± 1.86% and 47.55 ± 1.81%, respectively (*p* = 0.98 as determined by a Student’s *t*-test). We did not observe significant differences among the LE of Dox on ANCs and ReANCs (Supplementary Figure S2A). Dreis et al. (2007) have reported a minimum Dox LE of 69% following adsorption to albumin nanoparticles; however, this LE was reported when Dox loading was performed in water. In this study, the decreased solubility of Dox in PBS could contribute to a lower LE. Notably, despite the lower LE, we were able to tune the amount of Dox loaded onto ANCs and ReANCs to optimal therapeutic dosing levels based on the initial loading amount (Supplementary Figure S2B).

### Sustained Release of Dox From ANCs in a pH-Dependent Manner

**FIGURE 4:**
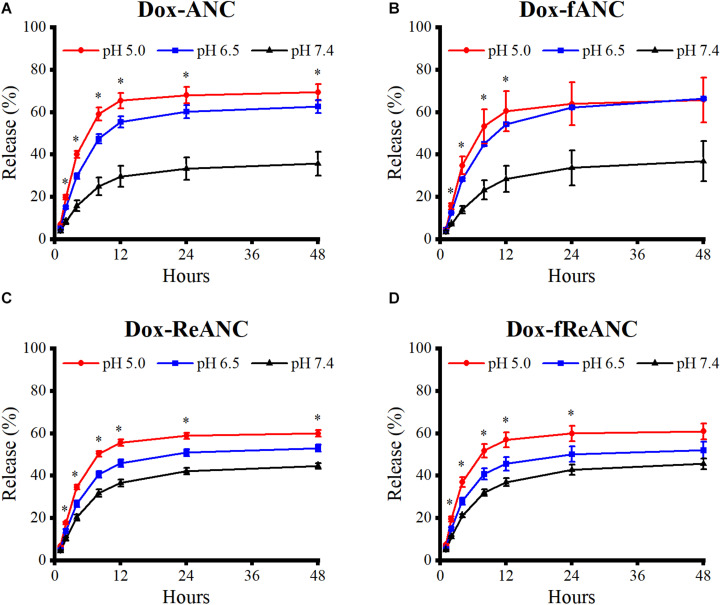
Pharmacokinetic release profiles of Dox from ANCs *in vitro*. Dox release from Dox-ANCs **(A)**, Dox-fANCs **(B)**, Dox-ReANCs **(C)**, and Dox-fReANCs **(D)** was evaluated at 37°C in PBS at varying pH (5.0, 6.5, and 7.4) via microdialysis. Particles were loaded into microdialysis devices, which were then placed in 1 mL of release buffer consisting of PBS of varying pH in 5 mL centrifuge tubes (*n* = 3). At each time point, the microdialysis device was moved into a new tube containing fresh release buffer. The amount of released Dox was a measure of fluorescence (Ex: 470 nm, Em: 595 nm) from the release buffer at each indicated time point. Data is expressed as mean ± SEM. Dox was released from nanoparticles at a sustained rate over the course of 48 h in a pH-dependent manner, as assessed by a one-way ANOVA. Addition of rare earth nanoparticle cores or AMD3100 did not affect the release profiles, as assessed by a two-tailed student’s *t* test.

One of the criteria for an effective drug nanocarrier or theranostic agent is that a sufficient amount of the drug is released and accumulated at the tumor site. Doxorubicin release profiles from ANCs *in vitro* were determined by dialysis of Dox-ANCs, Dox-fANCs, Dox-ReANCs, and Dox-fReANCs at pH 5.0, 6.5, and 7.4. Our findings showed that pH had an impact on the release of Dox from ANCs, with more rapid release occurring in acidic environments (Figure 4A), consistent with other Dox nanocarriers (Lin et al., 2017; Bi et al., 2018). In acidic conditions, similar to those that would be found in the tumor microenvironment, the amine group on Dox gets protonated, improving its solubility in solution and increasing its propensity to be released from the nanocarrier (Bi et al., 2018). Release profiles of Dox from Dox-fANCs were similar to Dox-ANCs (Figure 4B), indicating that functionalization with AMD3100 did not adversely affect the release of Dox. There was no significant impact on the release profiles when Dox -ReANCs and Dox-fReANCS were used; drug release from Dox-ReANCs (Figure 4C) and Dox-fReANCs (Figure 4D) followed similar pH-dependent behavior.

### *In vitro* Pharmacodynamic Analysis

**FIGURE 5:**
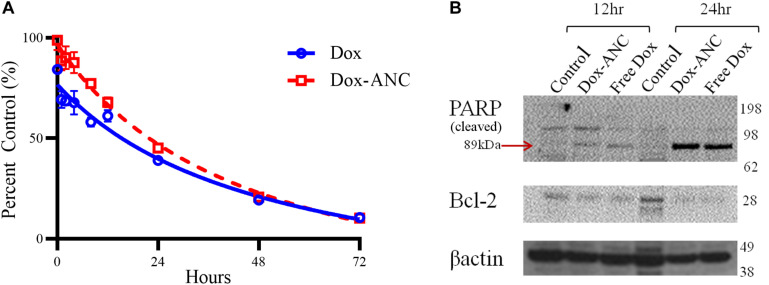
Dox-ANCs induce cytotoxicity through activation of cell-death pathways. MDA-MB-231 cells (10^4^ cells/well) were treated with an equivalent dose of 0.02 mg/mL Dox-ANCs or free Dox over 72 h. Viability was assessed via an MTT assay at times 0, 1, 2, 4, 8, 12, 24, 48, and 72 h of treatment, and the absorbance at 570 nm was normalized to untreated controls **(A)**. Data is expressed as mean ± SEM for *n* = 3. The cytotoxic effect of Dox-ANC was delayed in comparison to free Dox during the first 24 h. To assess the effect of Dox and Dox-ANCs on cellular pathways of cell death, an immunoblot was run from samples of MDA-MB-231 cells lysed after 12 and 24 h of treatment with Dox (Dox-ANCs or free Dox) or untreated. Qualitative analysis of the blots revealed elevated levels of cleaved PARP and decreased expression of Bcl2 in cells treated with Dox-ANC and free Dox compared to untreated control **(B)**. This indicates an active cell death process in response to the delivery of Dox.

Dox targets key DNA replication proteins, interfering with the cellular ability to properly divide and thus resulting in induction of cell death pathways, particularly apoptosis. We first evaluated the differences in viability of MDA-MB-231 cells following treatment with Dox-ANC and free Dox over a 72-h time course. Overall, there was little difference between Dox-ANC treated and free Dox treated cells (Figure 5A), suggesting that the therapeutic efficacy was maintained with the Dox-ANC formulation.

Next, pharmacodynamic changes as a function of apoptotic marker expression were assessed by immunoblot following treatment of MDA-MB-231 cells with either Dox-ANC or free Dox for 12 and 24 hours. Expression of Bcl2, a key anti-apoptotic protein, decreased in the presence of doxorubicin (both free Dox and Dox-ANC treated) compared to control cells (Figure 5B). PARP is an important nuclear enzyme that enables DNA repair mechanisms caused by chemotherapeutic drugs, and cleavage and subsequent inactivation of PARP will lead to increased cytotoxicity (Morales et al., 2014). We observed an increase in PARP cleavage (both free Dox and Dox-ANC treated), indicating an active cell death process.

### Tumor-Targeting Increased Cellular Uptake of Doxorubicin *in vitro*

**FIGURE 6:**
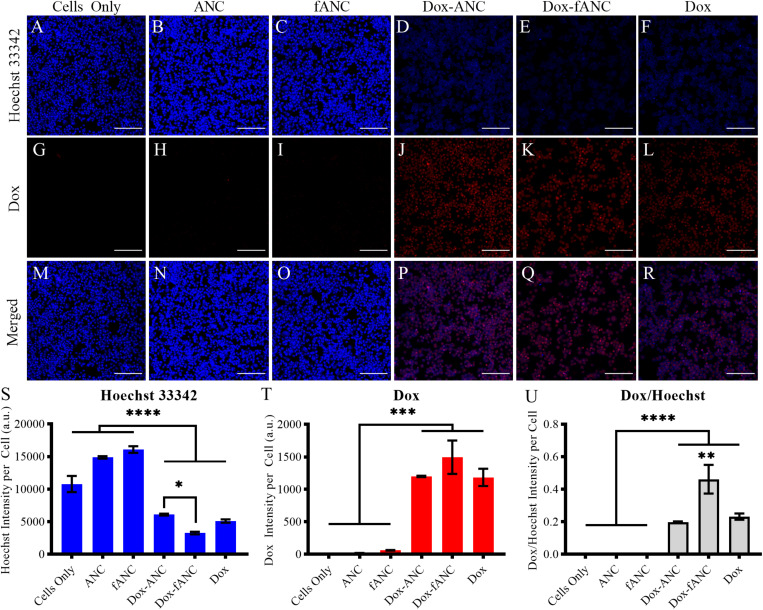
Increased Dox uptake *in vitro* by tumor-targeted nanoparticles. MDA-MB-231 cells were seeded into 96 well plates and treated with either culture media (cells only), ANCs, fANCs, Dox-ANCs, Dox-fANCs, or free Dox at an equivalent concentration of 0.02 mg/mL Dox and a 1x concentration of particles for 8 h. Cells were then fixed and stained with Hoechst 33342 to image the cell nuclei **(A–F)**. The natural fluorescence of Dox was used to image the uptake of Dox by cells **(G–L)**. Dox and Hoechst 33342 compete for binding spots on DNA; therefore, the Hoechst and Dox images were merged to observe colocalization in the cell nuclei **(M–R)**. The signal from the Hoechst stain **(S)** and Dox **(T)** was quantified from three distinct fields of view for each sample and normalized to the cell count. The effects of treatment and functionalization were assessed via a two-way ANOVA with a Tukey’s *post hoc* test to correct for multiple comparisons. The intensity of the Hoechst stain was significantly diminished in response to treatment. Inversely, the Dox signal was significantly greater in cells that received treatment. Since the decrease in Hoechst signal is due to intercalation of Dox to the DNA, the Dox/Hoechst ratio was assessed **(U)**. The Dox/Hoechst ratio was greatest in cells treated with Dox-fANCs, indicating increased uptake of Dox when a targeted nanocarrier is used for administration *in vitro*. Data representative of three independent experiments. Scale bar = 200 μm. Data in S-U are expressed as mean +SEM from triplicate samples. ^∗^*p* < 0.05, ^∗∗^*p* < 0.01, ^∗∗∗^*p* < 0.001, ^****^p < 0.0001.

Targeting has been shown to enhance cellular uptake of nanoparticles and also minimize off-site toxicity (Yameen et al., 2015; Peng et al., 2017). We investigated whether tumor-targeted Dox-ANCs would lead to increased uptake in tumor cells. We determined effective cellular uptake of Dox-ANCs and Dox-fANCs as a measure of Dox-associated fluorescence. Doxorubicin, subsequent to cellular uptake, exerts its anti-tumor effect by localizing in the nucleus and intercalating with cellular DNA (Zhao et al., 2018), and in concordance with this, we observed a significant decrease in Hoechst staining in cells treated with Dox-ANC and Dox-fANC (*p* < 0.0001) (Figures 6A–F,S). Additionally, Dox fluorescence intensity was significantly higher in treated cells (*p* < 0.0001) compared to untreated cells (Figures 6G–L,T). These observations, in combination, suggest that Dox accumulated and intercalated in the nuclei of cells. A correlation between increasing Dox and decreasing Hoechst fluorescence signal was shown by examining both fluorescence channels (Figures 6M–R) and normalizing the Dox fluorescence to the Hoechst signal (Figure 6U). The ratio of Dox to Hoechst was significantly greater in the treated groups compared to the control groups, and particularly in the Dox-fANC treated group compared to Dox-ANC (*p* = 0.0030) or free Dox (*p* = 0.0090). There were no significant differences between the Dox-ANC and free Dox group, suggesting that the nanoparticle formulation did not have an appreciable impact on Dox cellular uptake. Taken together, these results suggest that while delivering Dox in a nanoparticle formulation compared to free Dox did not impact cellular uptake or its pharmacodynamic profile, tumor-targeted ANCs improved efficacy of Dox *in vitro*.

### Anti-tumor Efficacy and Therapeutic Monitoring of Tumor-Targeted Dox-Loaded Nanoparticles *in vivo*

**FIGURE 7:**
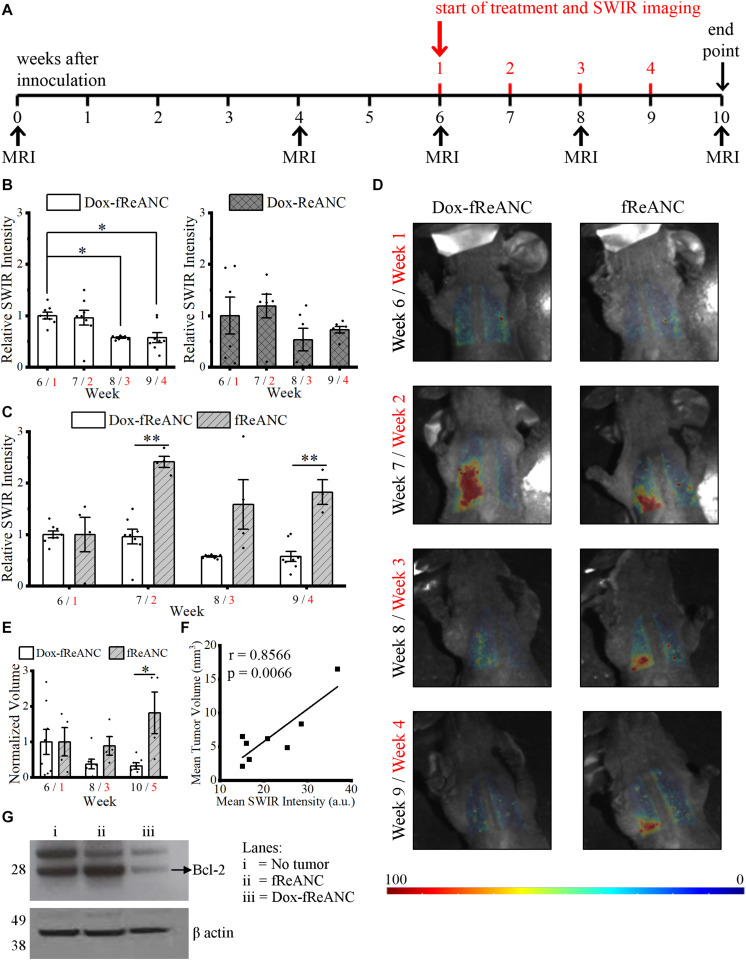
Theranostic rare earth-based nanoparticle mediated therapeutic drug monitoring in a mouse model of lung metastases. Mice were inoculated with human triple negative breast cancer cells (MDA-MB-231) via intravenous tail vein injection. Biweekly MRI was performed from inoculation until the end of the experiment. The first week of the four-week treatment started six weeks after inoculation, when tumors in the lungs were discernible by MRI **(A)**. Weeks after inoculation are presented in black text, and weeks after initiating treatment are presented in red text. Animals in the Dox treatment groups received weekly 150 μL injections of Dox-ReANCs (*n* = 6) or Dox-fReANCs (*n* = 8) at an equivalent dose of 2–2.5 mg/kg Dox. Tumor-bearing control mice received weekly injections of ReANCs (*n* = 5) or fReANCs (*n* = 4). Whole body SWIR imaging of the mice was performed at 24 h post-injection. To compare the effects of treatment among groups, data was normalized to the SWIR signal from week 1, and a two-way ANOVA was performed, followed by a *post hoc* Tukey’s test to correct for multiple comparisons. The SWIR signal was similar between mice that received Dox-ReANCs and Dox-fReANCs **(B)**. However, there was a significant decrease in SWIR emissions from the lungs in mice injected with Dox-fReANCs during the third (*p* = 0.0204) and fourth (*p* = 0.0176) week of treatment, as determined by a one-way ANOVA followed by a Tukey’s *post hoc* test, indicating that functionalization led to better therapeutic outcomes. There was a significant difference in the SWIR signals in response to treatment, as seen by the differences in quantified SWIR signal **(C)** and representative SWIR images **(D)** of the lungs of mice that received Dox-fReANCs and non-therapeutic fReANCs. There was an overall increase in the SWIR signal in mice that received fReANCs. There was also a significant decrease in SWIR signal over the course of treatment in mice treated with Dox-fReANCs compared to mice injected with fReANCs during week 2 (*p* = 0.0011) and week 4 (*p* = 0.0010). Changes in tumor burden in fReANC and Dox-fReANC treated mice were validated by volumetric analysis from MRI **(E)**, with a significant reduction in tumor volume following treatment compared to the untreated control (*p* = 0.0380). Mean SWIR signal from weeks 1 and 3 were found to correlate with the respective tumor volumes assessed by MRI **(F)**, with a Pearson’s r of 0.8566 (*p* = 0.0066). At endpoint, the lungs were harvested, homogenized, and lysed. An immunoblot was performed from the lung lysates and probed for Bcl-2 expression **(G)**. Protein levels of Bcl-2 were decreased in the lungs of mice treated with Dox-fReANCs when compared to untreated mice or non-tumor-bearing healthy controls. All data is expressed as the mean ± SEM. ^∗^*p* < 0.05, ^∗∗^*p* < 0.01.

Previous work by our lab has shown that functionalization of ReANCs with a targeting ligand not only improves retention of the nanoparticles at the lesion site, but also enables longitudinal imaging of micro-lesions and tumor progression *in vivo* (Zevon et al., 2015; Kantamneni et al., 2017). Here, we demonstrated the enhanced anti-tumor efficacy of tumor-targeted Dox-fReANCs compared to Dox-ReANCs in an established lung metastasis mouse model of human breast cancer (Ganapathy et al., 2010). The inoculation and treatment timeline is described in Figure 7A. Targeted Dox-loaded fReANCs showed a significant reduction in SWIR fluorescence during the fourth week (∼0.6-fold when normalized to week 1), but Dox-ReANC signal was not statistically different throughout the study (Figure 7B). This correlated with a decrease in tumor volume in the targeted Dox-fReANC animals compared to untreated controls, as assessed by MRI (Figure 7E). On the contrary, there was a slight, but not significant, increase in tumor volume of untargeted Dox-ReANC animals (Supplementary Figure S3). Furthermore, no significant difference in tumor volume was seen between the Dox-ReANC-treated animals or the controls. These results suggest that targeted particles can potentially improve the therapeutic outcome by enhanced retention at tumor site.

Similar to previous reports (Zevon et al., 2015), more SWIR signal was detected from fReANCs than from ReANCs as tumors progressed (Supplementary Figure S4A), indicating that improved targeting with functionalization is retained longitudinally. The therapeutic effect of increased retention at the lesion site was also evident through an increase in signal from non-therapeutic fReANCs as tumors progressed (indicative of a lack of tumor regression) compared to therapeutic Dox-fReANCs (2.5-fold, 2.8-fold, and 3.2-fold greater in fReANCs than Dox-fReANCs during weeks 2, 3, and 4, respectively) (Figure 7C). Similarly, but not to a significant extent, ReANCs produced more signal than Dox-ReANCs (Supplementary Figure S4B). Taken together, this data suggests that ReANCs are capable of at-lesion delivery of therapeutic payloads, and the therapeutic effect is improved when the theranostic nanocarriers are functionalized with a targeting ligand.

The theranostic potential of the particles, as a function of tumor regression, was assessed by comparing the ability of ReANCs to monitor therapeutic responses based on SWIR emissions, and findings were validated by MRI. Representative images of mice during weeks 1 through 4 of treatment are shown in Figure 7D. We demonstrated a significant decrease in SWIR emissions in mice treated with Dox-fReANC at week 3 post-treatment (*p* = 0.0204), and the trend continued to week 4 (*p* = 0.0176) (Figures 7B,C). Neither ReANC nor Dox-ReANC treated animals showed a significant change in SWIR emissions throughout the study (Supplementary Figures S4B,C). The decrease in SWIR emissions in Dox-fReANC treated animals correlated with decreases in tumor volume as assessed by MRI (Figure 7E). Notably, SWIR intensity was found to correlate with tumor volume with a Pearson correlation coefficient of *r* = 0.8566 (*p* = 0.0066) (Figure 7F). Further, decreased levels of the anti-apoptosis protein Bcl-2 were observed in lung lysates from mice treated with Dox-fReANCs compared to untreated mice and non-tumor-bearing mice (Figure 7G). Taken together, the data suggest that targeted theranostic particles, through increased retention at the tumor site, have enhanced anti-tumor efficacy and are able to monitor therapeutic outcomes in the lung metastasis model over a four week period.

### Theranostic ReANCs and fReANCs Do Not Accumulate in the Heart

A major drawback of anthracyclines like Dox is the high risk of systemic toxicity and cardiomyopathy, due in part to a lack of control on drug delivery. In order to elucidate the localization of Dox and their nanocarriers in the body, we imaged the heart and lungs, as well as organs of clearance such as the liver, spleen, and kidneys. SWIR imaging revealed that nanoparticles accumulated either in the lungs where there was tumor, or in the liver and spleen (Supplementary Figure S5), which are the common organs of clearance for nanoparticles. No signal was seen in the heart, indicating that ReANCs did not accumulate in the heart. This finding was further validated by fluorescent images of the organs *ex vivo*, where Dox fluorescence was seen in the lungs, liver, and spleen, but not in the heart (Supplementary Figure S6), suggesting that Dox-fReANCs are not likely to cause cardiotoxicity. Furthermore, there were no significant differences among the weights of mice during the course of the study (Supplementary Figure S7).

## Discussion

Current clinical approaches for TDM in cancer involve assessing pharmacokinetic outputs, such as plasma drug concentration (Alnaim, 2007; Bartelink et al., 2019), and anti-tumor efficacy based on tumor regression and progression-free survival rates (McMahon and O’Connor, 2009). However, in cancer, the vasculature around the target tumor site is complex, and tissue penetration of the drug is unique, playing a significant role in drug efficacy (Bartelink et al., 2019). Hence, theranostics offer a tailor-made therapy monitoring tool for individual patients to assess the efficacy of drugs at the target tumor site, allowing for early intervention and regimen alterations where needed. In particular, a theranostic with the promise of molecularly monitoring pharmacodynamic outputs would be valuable in advancing the field of tailor-made patient-centric therapy monitoring.

The prognosis of triple negative breast cancer (TNBC) remains poor, and due to a lack of targetable biomarkers, chemotherapy is the only systemic therapy widely available (Waks and Winer, 2019). Recently, atezolizumab with nab-Paclitaxel was approved as a combinatorial therapeutic for metastatic TNBC for a subpopulation of programed cell death ligand 1 (PD-L1)-positive patients with advanced metastatic breast cancer (Heimes and Schmidt, 2019; Narayan et al., 2020; Reddy et al., 2020). However, chemotherapeutic agents are often associated with a relatively low response rate (Lebert et al., 2018) and adverse side effects, such as cardiotoxicity in the case of Dox (Zhao et al., 2018; Cranmer, 2019; Zhang et al., 2020). There is a critical need for monitoring TNBC therapy response in real time to provide altered regimen options early. In this study, we demonstrated the potential of a tumor-targeted theranostic nanoparticle that can not only deliver therapeutic payload to the tumor, but also provide an optical-based metric for response at the lesion site through SWIR emission intensities at a sub-tissue level (Naczynski et al., 2013; Zevon et al., 2015; Kantamneni et al., 2017).

ReANCs are versatile nanocarriers, owing to the presence of drug binding pockets and the presence of functional groups for payload and targeting ligand adsorption and conjugation (Frei, 2011; Zsila et al., 2011; Liu and Chen, 2016). We engineered a theranostic formulation through stepwise integration of a tumor-targeting ligand (AMD3100) and therapeutic payload (Dox) onto the surface of ReANCs (Figure 2). One of the advantages of drug nanocarriers is their ability to deliver a drug in a spatiotemporally controlled manner such that adverse toxicities can be avoided, and the drug can be delivered directly to the target tumor site for maximal efficacy by virtue of abnormal vasculature and EPR within the tumor space (Lee and Yeo, 2015). Drug release kinetics performed via dialysis showed a pH- and time-dependent release of Dox from albumin nanocomposites (Figure 4) in alignment with previous studies (Lin et al., 2017; Bi et al., 2018). We then examined the therapeutic efficacy of the particles as nanocarriers by comparing the treatment of cells with Dox-ANC and free Dox in solution. We noticed a decreased rate of cell death by Dox-ANCs during the first day of treatment (Figure 5A), and this was in correlation with the release of active Dox from the nanocarrier where we saw release profiles reach maximum and plateau after 12 hours (Figure 4). Anti-tumor efficacy of Dox nanocarriers was compared with free Dox through qualitative analysis of pharmacodynamic markers (Bcl-2 and PARP; regulated by the apoptotic pathway), which showed little difference between the groups (Figure 5B). Specifically, Bcl-2, a protein associated with the suppression of apoptosis, exhibited decreased expression in the presence of Dox when compared to untreated cells (Figure 5B) or animals (Figure 7G). The role of Dox in promoting apoptosis was further demonstrated by the increased cleavage of PARP in cells treated with Dox when compared to untreated cells.

Targeted nanoparticles have been suggested to improve the therapeutic efficacy of the drugs they carry (Ma et al., 2019). Previously, we have shown that particles functionalized with AMD3100 have enhanced retention in CXCR4-expressing tumor cells both *in vitro* and *in vivo* (Zevon et al., 2015; Kantamneni et al., 2017). Similarly, we found that cellular uptake of tumor-targeted Dox-ANCs was higher as assessed by DNA intercalation of Dox. We demonstrated a significant decrease in DNA-binding Hoechst fluorescence in cells treated with Dox-fANCs compared to those treated with Dox-ANCs or free Dox. This correlated with an increase in Dox fluorescence in these cells, suggesting that tumor targeting enhances anti-tumor efficacy of Dox in the nanocarrier system (Figure 6).

An ideal theranostic would combine a therapeutic with a diagnostic that can molecularly target and deliver the payload at the target site while enabling real-time monitoring of therapeutic outcomes. ReANCs, as diagnostic agents, have been shown to allow for deep tissue imaging of tumor micro-lesions with a high signal-to-noise ratio (Naczynski et al., 2013; Zevon et al., 2015; Kantamneni et al., 2017). Additionally, ANCs and ReANCs have been shown to allow for therapeutic loading of small molecule drugs and targeting ligands, including peptides and antibodies (Naczynski et al., 2010, 2013; Cui et al., 2013; Zevon et al., 2015; Kantamneni et al., 2017). In a proof-of-concept model for breast cancer lung metastases, we showed that there was an increased anti-tumor effect in mice treated with Dox-fReANCs as evidenced by a significant decrease in SWIR signal, which correlated to tumor burden (Figure 7). The changes in SWIR signal intensity with Dox treatment was correlated and validated using MRI (Figure 7E). Notably, we observed no significant changes in SWIR signal in animals treated with Dox-ReANCs or in their tumor burden (Supplementary Figure S4), as validated by MRI.

In conclusion, we have developed SWIR-emitting theranostic nanoparticles capable of (1) targeted delivery of Dox to the tumor site and (2) imaging changes in the tumors in real time as a response to therapy. ReANCs loaded with Dox were capable of eliciting an anti-tumor response through tumor regression in a lung metastatic mouse model of human TNBC, as quantified by changes in SWIR fluorescence intensity and validated through MRI volumetric analysis. Importantly, the anti-tumor effect was markedly enhanced with tumor-targeted theranostic nanoparticles, suggesting the possibility for assessing effects of anti-cancer drugs in real time in different molecular subtypes of the tumor. Multispectral ReANCs with distinct spectra have recently been shown to molecularly discern distinct tumor subpopulations (unpublished data), and future studies will explore the ability to use ReANCs to track the response of combination chemotherapy, immunotherapy, and targeted therapy in distinct molecular tumor subsets.

## Code Availability Statement

The MATLAB image processing scripts used for this study are available on Github (https://github.com/markpierce50/Frontiers---2020).

## Data Availability Statement

The datasets generated for this study are available on request to the corresponding authors.

## Ethics Statement

The animal study was reviewed and approved by Institutional Review Board for the Animal Care and Facilities Committee of Rutgers University.

## Author Contributions

JS, AG, PM, and VG contributed to the conception and design of the study. JS, AG, and VG performed to the *in vivo* experiments. JS, AG, RP, and AS performed to the *in vitro* experiments. JS, AG, RP, AS, PM, and VG wrote the manuscript. SH, XZ, RR, and MT designed and synthesized rare earth nanoparticles. CBM and MP contributed to design of the *in vivo* SWIR imaging system. CBM, MB, and MP contributed to the writing of MATLAB code. All authors contributed to manuscript revision, read, and approved the submitted version.

## Conflict of Interest

The authors declare that the research was conducted in the absence of any commercial or financial relationships that could be construed as a potential conflict of interest.

## Footnotes

1 https://imagej.net/Fiji
