# Supplementary figures and images for: Shortwave Infrared-Emitting Theranostics for Breast Cancer Therapy Response Monitoring
Source: Front Mol Biosci. 2020 Oct 6;7:569415. doi: 10.3389/fmolb.2020.569415 (PMC7575924; doi:10.3389/fmolb.2020.569415)

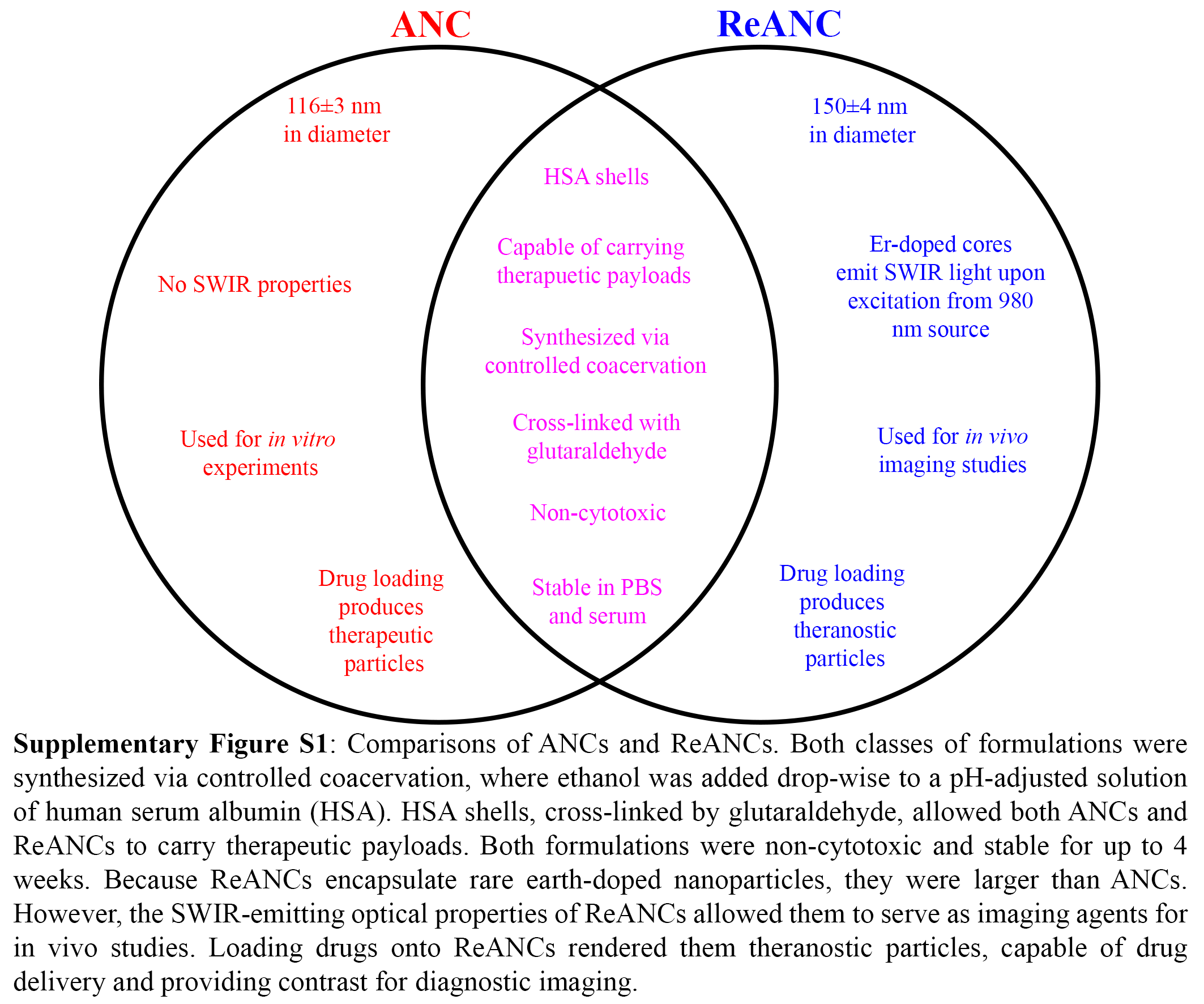

Supplement: Supplementary file 1 [file Image_1.TIF]

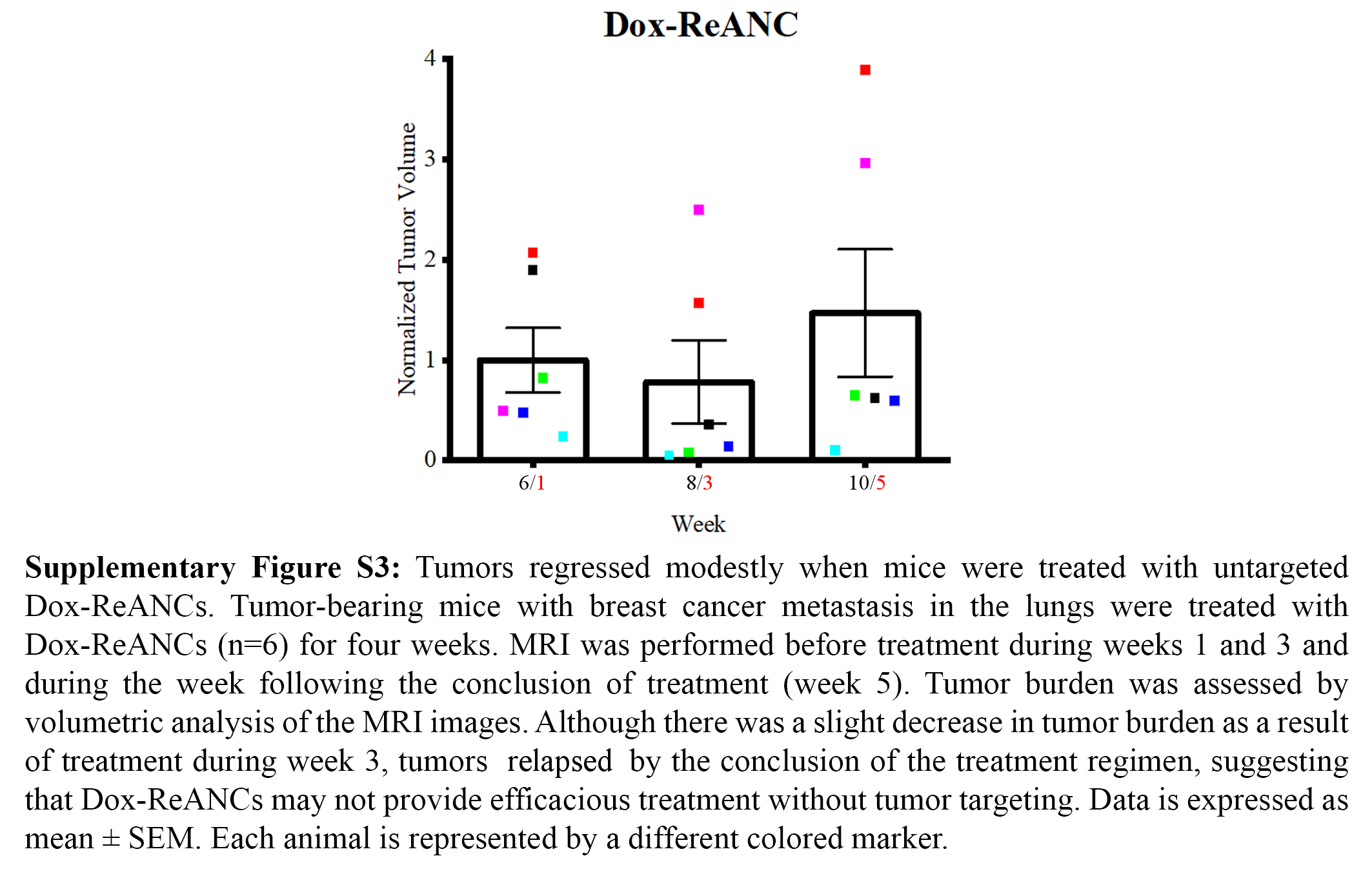

Supplement: Supplementary file 3 [file Image_3.TIF]

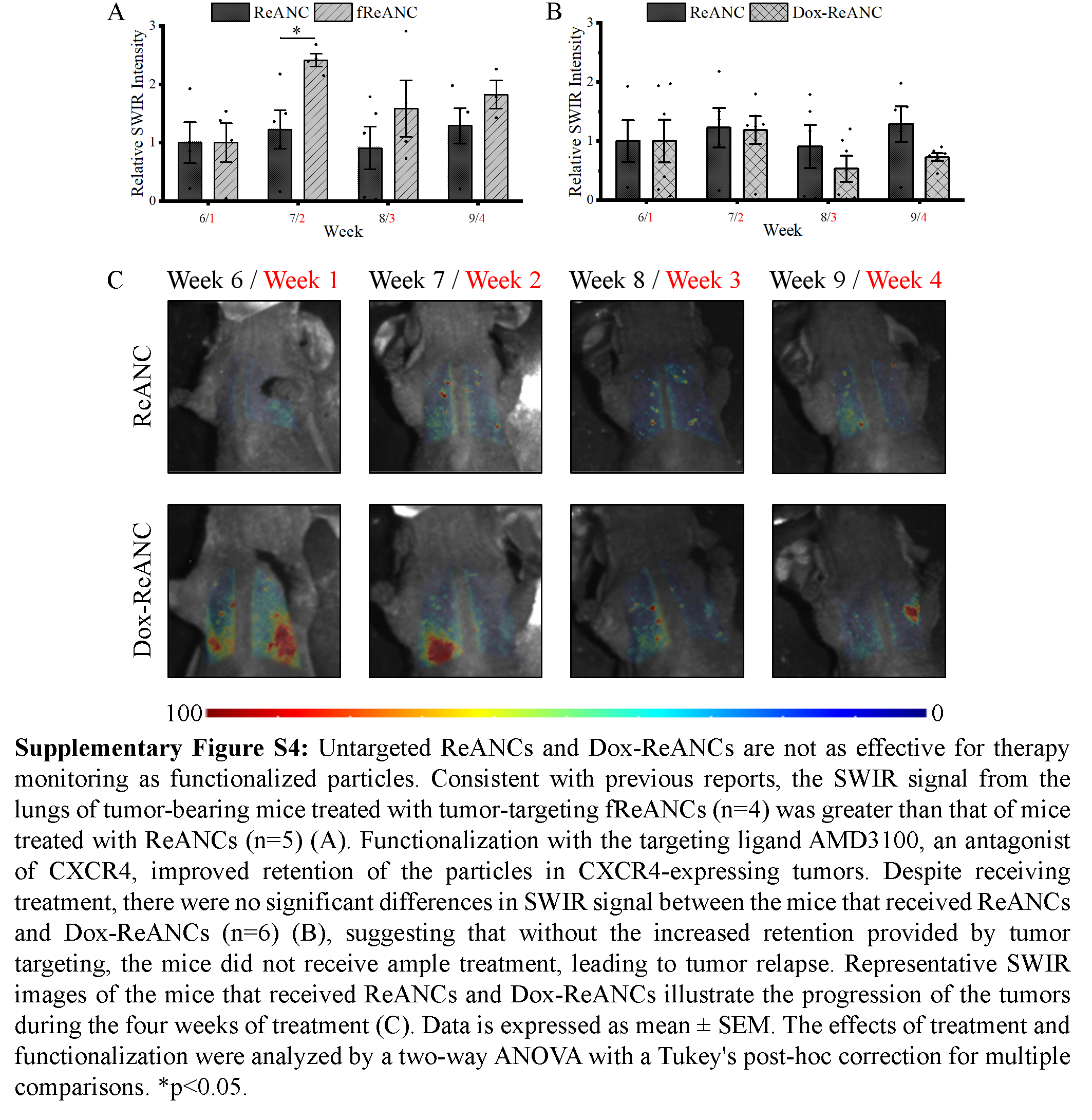

Supplement: Supplementary file 4 [file Image_4.TIF]

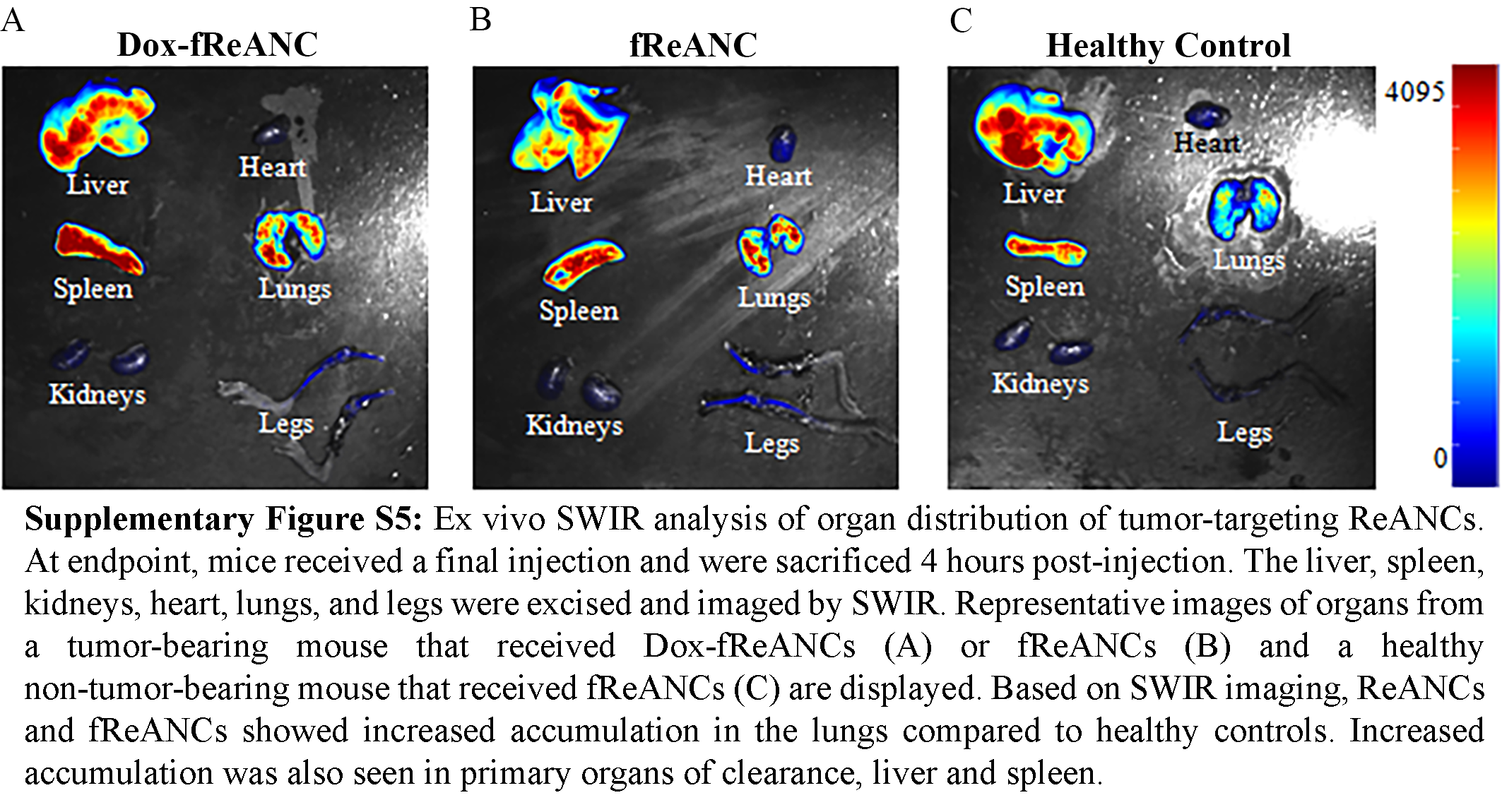

Supplement: Supplementary file 5 [file Image_5.TIF]

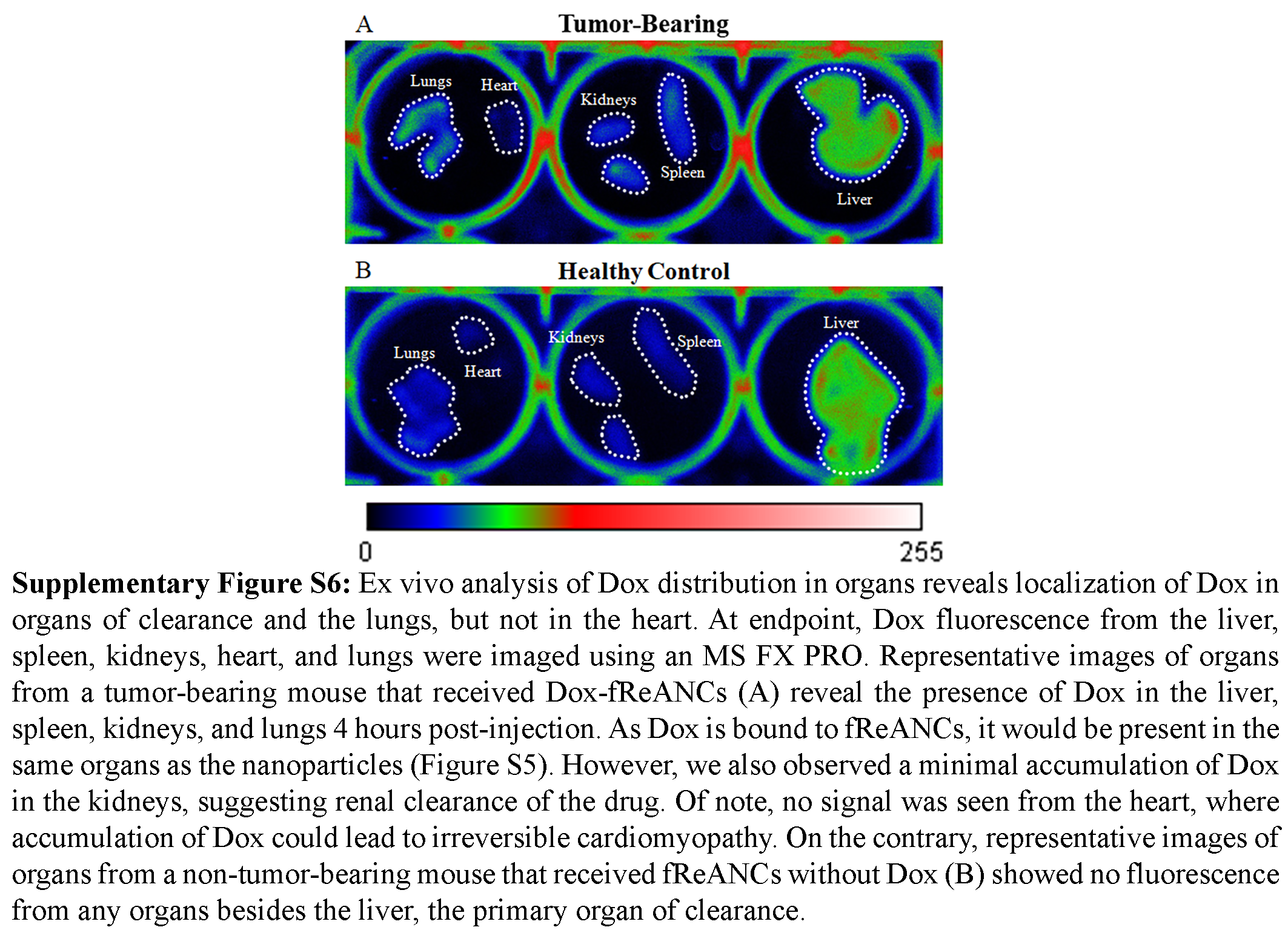

Supplement: Supplementary file 6 [file Image_6.TIF]
